# The Superfund Research Program Analytics Portal: linking environmental chemical exposure to biological phenotypes

**DOI:** 10.1038/s41597-023-02021-5

**Published:** 2023-03-21

**Authors:** Sara J. C. Gosline, Doo Nam Kim, Paritosh Pande, Dennis G. Thomas, Lisa Truong, Peter Hoffman, Michael Barton, Joseph Loftus, Addy Moran, Shawn Hampton, Scott Dowson, Lyndsey Franklin, David Degnan, Lindsey Anderson, Anne Thessen, Robyn L. Tanguay, Kim A. Anderson, Katrina M. Waters

**Affiliations:** 1 Pacific Northwest National Laboratory, Richland, WA USA; 2 Oregon State University, Corvallis, WA USA; 3 University of Colorado Anschutz Medical School, Denver, CO USA

**Keywords:** Pollution remediation, Environmental impact

## Abstract

The OSU/PNNL Superfund Research Program (SRP) represents a longstanding collaboration to quantify Polycyclic Aromatic Hydrocarbons (PAHs) at various superfund sites in the Pacific Northwest and assess their potential impact on human health. To link the chemical measurements to biological activity, we describe the use of the zebrafish as a high-throughput developmental toxicity model that provides quantitative measurements of the exposure to chemicals. Toward this end, we have linked over 150 PAHs found at Superfund sites to the effect of these same chemicals in zebrafish, creating a rich dataset that links environmental exposure to biological response. To quantify this response, we have implemented a dose-response modelling pipeline to calculate benchmark dose parameters which enable potency comparison across over 500 chemicals and 12 of the phenotypes measured in zebrafish. We provide a rich dataset for download and analysis as well as a web portal that provides public access to this dataset via an interactive web site designed to support exploration and re-use of these data by the scientific community at http://srp.pnnl.gov.

## Background & Summary

The Comprehensive Environmental Response, Compensation, and Liability Act (CERCLA), also known as the Superfund, has been credited with cleaning up over 800 toxic waste sites across the United States since it was enacted in 1980^1^. Despite the aggressive clean-up activity, polycyclic aromatic hydrocarbons (PAHs) continue to raise concern and serve as the risk driver for remediation at 20% of all Superfund sediment sites^2^. While it has been established PAH contamination still exists at these sites and can affect human health in nearby communities, the specific impact that results from exposure to various PAH contaminants is not well characterized. Toward this end, we introduce the SRP Analytics Compendium, an online resource that captures chemical measurements from Superfund site samples and links them to their potential biological impact in humans using model organism data, available at http://srp.pnnl.gov for interactive browsing and https://data.pnnl.gov/group/nodes/dataset/13337 for download and analysis.

The scientific community has identified more than 100 parent PAH compounds and numerous substituted and degradation products of PAHs. While parent compounds arise from industrial processes and extraction and burning of fossil fuels in industrial countries^3^, lower-temperature combustion activities, such as smoking tobacco or burning wood, generate lower-molecular-weight PAHs. Conversely, higher-temperature industrial processes and wildfires typically generate higher-molecular-weight PAHs^4^. Substantially higher concentrations of PAHs exist in the soil, water, and outdoor air in Asia, Africa, and Latin America than in Europe, Australia, the U.S., and Canada^3^, but as we and others have demonstrated, PAHs travel long distances to impact human exposures^5–8^. Most importantly, PAHs typically exist in complex environmental mixtures^4,9^. PAHs have been shown to cause cancer^10^, impede normal development^11,12^, suppress the immune system^13,14^, and target the neurological systems^15^. Furthermore, there is a strong association between maternal exposure to PAHs and paediatric neuro-behavioural problems: early-life PAH exposures have been shown to have an effect on childhood cognition and attention deficit disorders^16–19^, as well as obesity and asthma^20^.

The Oregon State University Superfund Research Program focuses on the identification of PAHs in the environment and characterizing their toxicity. We have pioneered the development of passive sampling devices to measure chemical mixtures at various Superfund sites around the Pacific Northwest^21–25^. We have also demonstrated the utility of the embryonic zebrafish model system for high throughput screening of chemicals for hazard assessment^26–28^. By collecting real-world chemical mixtures at Superfund sites and evaluating their toxicity in the zebrafish model, we supply regulatory agencies with actionable information and tools to prioritize sites for remediation and evaluate the effectiveness of remediation strategies.

To facilitate the sharing of data we have collected on PAHs in the environment, we introduce the Superfund Research Program Analytics compendium, a harmonized, open data resource that enables members of the larger research community to browse environmental samples from diverse Superfund sites, and to identify their chemical composition as well as observe their impact on zebrafish using 18 different morphological and behavioural endpoints. Of the these 18 endpoints, 12 are displayed on the web portal. The 12 endpoints include the most commonly measured endpoints and 6 summary endpoints to allow for ease of comparing chemical bioactivity. We also provide summaries of the transcriptome data describing the effect of these chemicals on human cell lines from the Carcinogenome Project^29^ as well as links out to the Carcinogenome Project web application. In addition to sharing the processed data files^30^, we also have provided a browsable interface by which scientists can query and interact with the data^31^. Our system is built using a containerized data processing pipeline so that additional data types can be added to our repository for future release.

## Methods

**Fig. 1:**
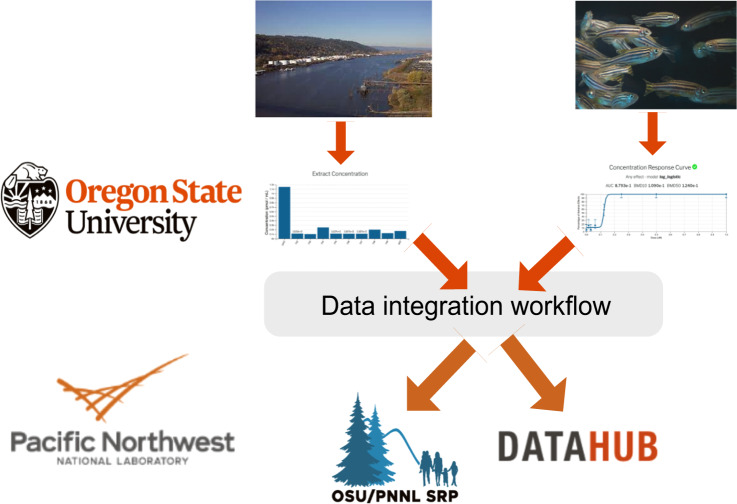
Overview of data collection and analysis pipeline to generate SRP Analytics Compendium.

Our approach integrates data from environmental sample measurements together with data from zebrafish activity using a containerized workflow to create reproducible results across years of sample collection and experimental measurement, depicted in Fig. 1.

In short, environmental sample extracts were collected via low-density polyethylene (LDPE) passive sampling devices (PSDs) from diverse Superfund sites in the United States. The composition of these environmental sample extracts was measured (top left) by deploying low-density polyethylene (LDPE) passive sampling devices (PSDs), and quantifying the samples on a gas chromatograph (GC) for 62 PAHs which were then evaluated in zebrafish to ascertain any morphological defects caused by the PAHs^27,32^. We then expanded the chemical measurements in zebrafish to over 400 other chemicals summarized in Table 1. Chemicals were classified into one of ten classes (or left unclassified): anilines, dioxins and furans, halo ethers, oxygenated polycyclic aromatic hydrocarbons (OPAH), organophosphorus flame retardants (OPFR), polycyclic aromatic hydrocarbons (PAH), polybrominated biphenyls (PBB), polybrominated diphenyl ethers (PBDE), polychlorinated biphenyls (PCB), and phenols. Those that have not been annotated yet, or do not fall into one of the 10 classes, were labelled as ‘Unclassified’. The data from the environmental sample extracts as well as the individual chemicals were then processed via an analytical pipeline described below to a shared database, which is then showcased in the SRP Analytics Portal and shared on DataHub.

**Table 1:** Summary of chemicals by class, including the total number of chemicals in that class, the number of environmental samples that measured that chemical, as well as numbers of endpoint measurements that passed quality control and are included in the portal.

Chemical Class	Total chemicals in class	Chemicals detected in samples	Endpoint calculation for samples containing chemical	Chemicals with endpoint calculations	Endpoint Calculations for chemicals in class
Anilines	43	0	0	43	88
Dioxins and Furans	50	2	36	50	68
Halo Ethers	15	0	0	15	15
OPAH	22	12	28	21	138
OPFR	6	3	11	6	33
PAH	99	71	172	98	823
PBB	31	0	0	31	31
PBDE	62	0	0	61	88
PCB	208	2	2	207	207
Phenol	98	7	49	98	197
Unclassified	1846	86	72	1238	5191

Some chemicals were not measured in the samples or measured below the level of detection in the environmental samples and are therefore not in the portal.

The result, depicted in Table 1, is a collection of chemicals, environmental samples (extracts), and the number of endpoint measurements calculated to indicate the resulting impact each chemical had on the developing zebrafish.

### Passive sampling of superfund research sites

**Fig. 2:**
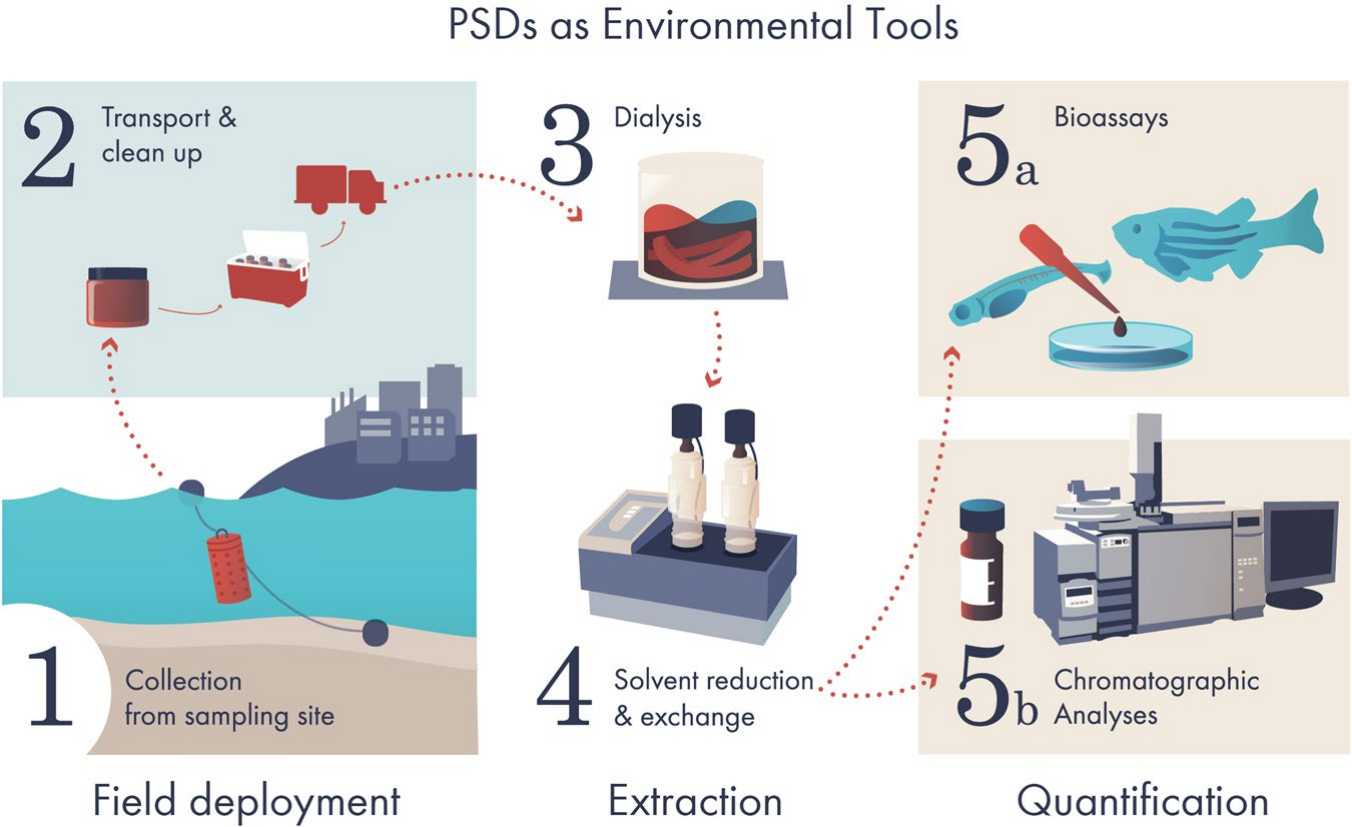
Overall schema for environmental sample extraction and quantification.

We used previously developed low-density polyethylene (LDPE) passive sampling devices (PSDs) to measure PAHs at Superfund research sites^33^, summarized in Fig. 2. For these data, LPDE PSDs were prepared as described previously^21^, using 2.5 cm wide × 70 µm thick LDPE tubing (Brentwood Plastics, St Louis, MO). Briefly, prior to construction, PSDs were cut to length, loosely rolled, placed in glass jars, immersed in Optima grade hexanes (Fisher, Fairlawn, NJ), and placed on a low-speed orbital shaker for 48 h with two changes of solvent. After the solvent was decanted, clean PSDs were dried under vacuum at room temperature until the residual solvent is removed. PSDs were then heat-sealed on each end with a small loop at the midpoint. Finished PSDs measured 100 cm in length.

Samplers were deployed by passing a steel ring through the centre loop on the PSD and suspending a “jellyfish” of 40 strips at the desired depth in the water column using weights and a buoy system. In many studies, co-deployed PSDs with performance reference compounds (PRCs) were used to calculate local free-water concentrations (C_free_). Because of the presence of PRCs, these samples were not appropriate for toxicological studies.

After deployment, PSDs were recovered, rinsed in the field to remove any biofouling, and returned to a metal canister for transportation to the lab and stored at −20 °C. LDPE strips were initially cleaned in the laboratory by light scrubbing and sequential washing in 1 N HCl and 18 MΩ-cm water, followed by two rinses with isopropanol, then dried and stored at −20 °C in batches of five strips per amber glass jar until extraction.

For extraction, the PSDs were dialyzed twice at room temperature with n‐hexane. Surrogate standards were added pre-extraction to quality control and performance reference compound‐containing samples, before the first addition of n‐hexane. No surrogate standards were added to samples intended for zebrafish analysis.

Extraction volume was quantitatively reduced to 1 mL using Turbo Vap closed-cell concentrators (Biotage) and small volume blow-down under a gentle stream of nitrogen. Concentrated extracts were transferred to amber glass vials, combining extracts from the same sampling site such that 8 mL represent 40 LDPE strips, hereby referred to as an environmental sample. Samples were stored at –20 °C. Prior to zebrafish exposure, environmental samples and fractions of those samples were evaporated to dryness under a gentle nitrogen stream and reconstituted in an equal volume of DMSO.

### Zebrafish biological measurements

The zebrafish model has become a common animal model for developmental toxicity studies as it is highly amenable to high-throughput animal screening^27,34,35^ due to the high degree of genetic and physiological similarity between zebrafish and other vertebrates. Because the embryos develop externally and are optically clear, we can carry out detailed morphological studies in our screens.

**Fig. 3:**
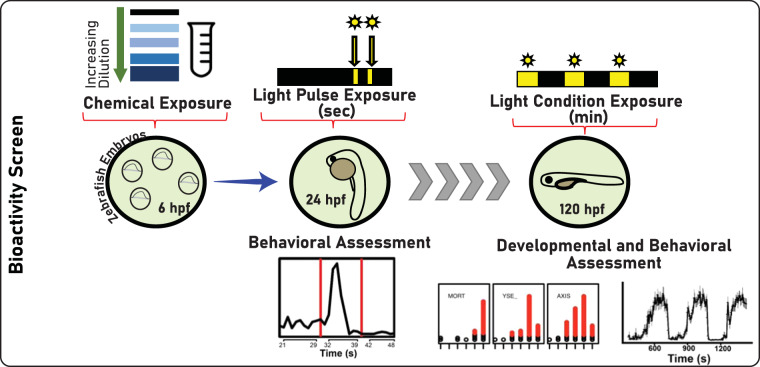
Depiction of zebrafish bioactivity screen.

The developmental toxicity experimental design used to generate the data in the SRP analytics compendium is described in Geier *et al*.^27^ and illustrated in Fig. 3. Briefly, Tropical 5D wildtype adult zebrafish were housed at a density of 1,000 fish in 100-gallon tanks kept on a 14-h light/10-h dark photoperiod. Embryos were collected and staged prior to dechorionation at 4 hours post-fertilization (hpf). At 4 hpf, the chorions were enzymatically removed and placed into individual wells of a round-bottom 96-well plate filled with 100 µL of embryo media. Endpoints were then mapped to existing ontologies^36^ or merged to form summary endpoints, described in Supplemental Table 1. Of the individual and summary endpoints, we selected 12 to display in the SRP Analytics portal, depicted in Table 2.

**Table 2:** Description of Zebrafish endpoints that are available on the portal as defined in Thomas *et al*.^37^ and ontology identifiers where available.

Endpoint labels	Short Name	Description	Ontology ID
MO24	Mortality at 24 hours	Death occurred shortly after chemical dosing to 24 hpf.	ZP:0000306
CRAN	Craniofacial	Periocular edema, abnormally small or missing eye(s), misshapen snout and protruding jaw form a highly correlated suite of malformations simplified to ‘craniofacial defects.’	ZP:0000943; ZP:0007203
AXIS	Axis	The body axis is visibly curved, either concave or convex, where control animals are straight.	ZP:0005012
EDEM	Edema	Distended clear region around the heart and/or yolk sac or top of the head with a lumpy appearance of swollen tissue.	ZP:0002060
LTRK	Lower Trunk	Shorter than normal body from the posterior of the yolk sac to the caudal fin, abnormal caudal fin	ZP:0003437; ZP:0010405
BRN	Brain	Brain region abnormal in appearance based on color or size.	ZP:0001601; ZP:0000100; ZP:0008625
ANY_MORT	Any effect except mortality	Any morphological abnormality not including mortality	
ANY120	Any effect in 5 days	Any abnormality or death within 5 days (combination of all measurements at 24 hr and 5 days)	
ANY24	Any effect in 24 hours	Any abnormality or death at 24hrs (aggregation of MO24, DP24, SM24 and NC24)	
TOT_MORT	Total Mortality	Total mortality (aggregation of MO24 and MORT)	
AUC1	5 day total movement	Behavior-total movement in dark minus total movement in light for the first light/dark cycle	ZP:0012599
MOV1	5 day behavior transition	Behavior-transition from light to dark for the first light/dark cycle	ZP:0012599

Some end points summarize a combination of other endpoints.

The samples used in these studies were provided in 100% Dimethyl Sulfoxide (DMSO), a solvent that induced no adverse effects on developing zebrafish up to 1% vol/vol. The chemicals were verified to be soluble in DMSO and then added by using a Hewlett Packard D300e and normalized to 1% DMSO (vol/vol). For the individual chemicals, stocks were provided at 20 mM, and the maximum test concentration was 50 µM, while the environmental samples were assessed at a maximum of 1% of the sample. Adverse morphological defects were assessed at two-time points: 24 and 120 hpf. At 24 hpf, mortality, developmental progression, spontaneous movement, and notochord distortion was assessed in each embryo. At 120 hpf, additional morphological endpoints were measured, including mortality, yolk, and pericardial edema, bent or curved bent body axis, abnormal eye, snout, jaw or brain, truncated body, response to touch, and failure of swim bladder to inflate (Supplemental Table 1). The data is recorded in a custom zebrafish laboratory information management system as binary incidence. Larval behaviour was assessed in the embryos using a photomotor response assay (LPR) at 120 hpf. The larvae are free-swimming and the total movement (swim distance) under multiple light- > dark transitions. The larvae in 96-well plates were tracked for 24-minutes in a Viewpoint Zebrabox (Montreal, CA). The light cycle consists of 3 minutes visible light and 3 minutes I.R. (dark), with movement being classified using two metrics – AUC and MOV – as described previously^37^.

To facilitate interpretability of the data and comparison with the Carcinogenome project, we chose 12 endpoints to visualize in the portal, depicted in Table 2. For example, the Muscle (MUSC) endpoint was not displayed but is incorporated in the ‘Any effect except mortality’ (ANY_MORT) endpoint. Furthermore, we compressed some of the mortality endpoints that made it difficult to compare across other datasets. However, all endpoints in Supplemental Table 1 are released in our data package, regardless of whether they are shown on the portal.

### Analyte processing and dose-response curve fitting

**Fig. 4:**
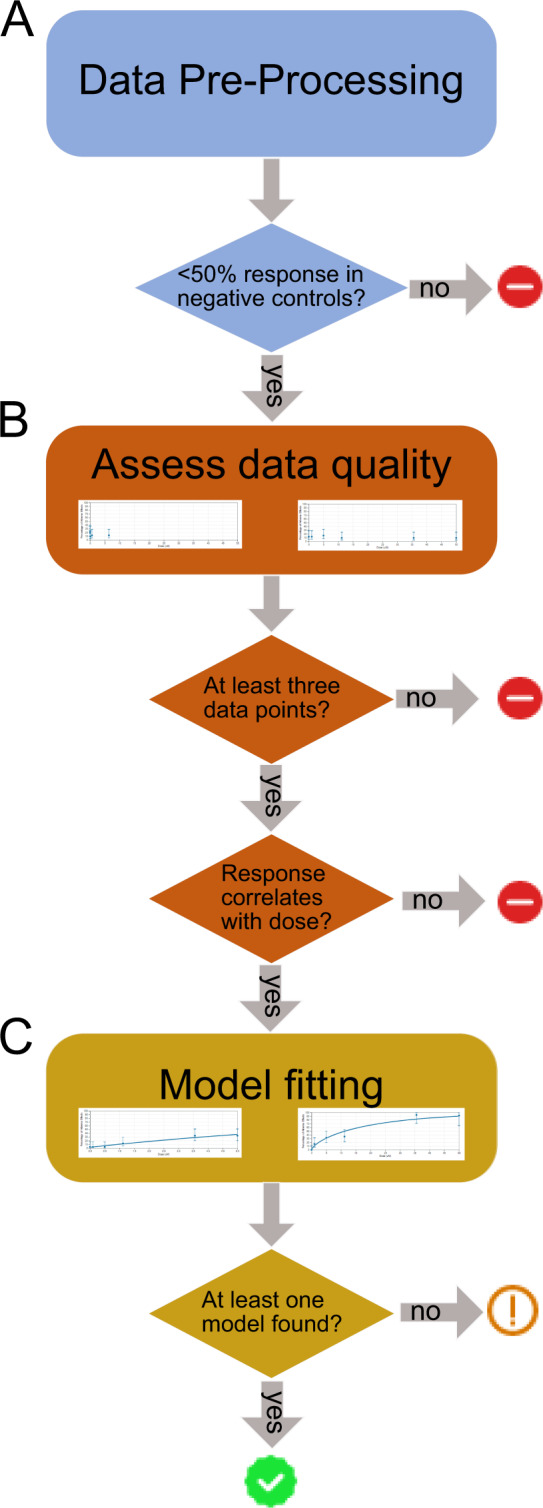
Data analysis pipeline described in three steps with depiction of quality control badge applied on the SRP data portal. (**A**) Data is pre-processed to ensure that there are appropriate negative controls. (**B**) Data is then evaluated to ensure there are at least 3 data points and that the curve goes in a stable direction. (**C**) Many models are attempted, if at least one model is identified the data receives a green check mark.

Determination of summary statistics in dose-response data is complex, with many competing algorithms and tools available. To rectify this, we have built a comprehensive workflow to assess the impact of the chemicals and environmental samples on the zebrafish^37^. This workflow was updated and standardized for release as part of the superfund research analytics portal at http://github.com/pnnl-compbio/srpAnalytics and is described in Fig. 4. The processing is broken down into three steps: data pre-processing (Fig. 4A), dose-response quality check (Fig. 4B) and dose-response curve-selection (Fig. 4C). The end goal is a quality control (QC) score of the data as well as benchmark dose (BMD) statistics if the QC score was deemed **Moderate** or **Good** as described below.

In the data pre-processing step (Fig. 4A), the binary morphological response data for individual zebrafish at each endpoint (0: no response observed, 1: response observed) is transformed to a standard dose-response data format by aggregating all wells (typically 32 wells) for each unique condition (e.g. pair of chemical ID and end-point). To increase the statistical power for separating the background effects from chemical effects, plates with more than 50% of the total embryos in negative control wells (vehicle only) that have observed responses are removed from the analysis. The combined counts from wells in the remaining plates are then used to obtain a fractional response for each concentration by dividing the number of wells with responding embryos (each well has one embryo) by the total number of wells with living embryos per each condition.

The dose-response data quality check (QC) removes data that are not appropriate to fit a curve model. First, it removes pairs of chemical ID and endpoint that have less than three concentration groups. Second, it assesses the curve quality (e.g. whether the response to each dose positively correlates with the concentration itself) and if the Spearman correlation between dose and response is below a threshold of 0.2 a curve is not fit. In both cases, a QC score of **Poor** is assigned, and no model is fit **(**Fig. 4B**)**.

The curve-selection step is applied to all dose-response data with a QC score that is not **Poor** as depicted in Fig. 4C. The data is fit to eight standard dose-response models for dichotomous data using loglikelihood optimization of model parameters: logistic, gamma, Weibull, probit, log-logistic, log-probit, multistage, and quantal linear. For each successfully converged optimization of model parameters, the fit quality is assessed by computing a chi-squared value and the associated p-value, the Akaike Information Criterion (AIC) value, and the scaled residuals. If a single model proves to be optimal, the curve fit is scored as **Good**. If there are multiple to choose from, the curve fit is scored as **Moderate** and the model with the lowest AIC is selected. A benchmark dose (BMD), is calculated from the curve to represent a dose or concentration that produces a predetermined change in the response rate of an adverse effect^38^, which is currently the US EPA-preferred dose-response assessment method. We calculate the concentration at which a 10% change in the incidence of the zebrafish phenotype is observed with chemical exposure relative to the control group (BMD_10_), as a point of departure to guide chemical risk assessment^37^. Furthermore, a benchmark dose lower bound (BMDL), which is the corresponding lower limit of a one-sided 95% confidence interval on the BMD, is also calculated by optimizing the profile likelihood for the BMD_10_ value for each model. Following the EPA guidance document^39^, the best model is selected based on the fit-quality metrics.

## Data Records

This resource compiles three different types of data into two distinct resources: the SRP analytics portal^31^ and a downloadable data package^30^ that contains eight distinct data files and one data dictionary (*v1Schema.xlsx*): extract composition and environmental measurements distinct scientific measurements (chemicalsByExtractSample.csv) measurements of the chemicals and environmental sample mixtures on zebrafish (chemdoseResponseVals.csv, chemSummaryStats.csv,chemXYcoords.csv, envSampleResponseVals.csv, envSampleSummarystats.csv, envSampleXYcoords.csv), and statistics describing number of genes differentially expressed upon treatment by chemicals on human cell lines (sigGeneStats.csv) as mined from the Xposome portal at https://montilab.bu.edu/Xposome/. The data files are shared on the PNNL DataHub^30^, with the schema for each file documented on our GitHub site https://github.com/PNNL-CompBio/srpAnalytics.

## Technical Validation

Here we describe the validation of data quality for the environmental sample chemical measurements and the zebrafish data.

### Measuring technical quality of passive samples

We quantified 62 PAHs on a modified Agilent 7890 gas chromatograph (GC) coupled to an Agilent 7000 triple quadrupole mass spectrometer (MS/MS)^40^. The internal standard, perylene-d12, was added to each sample or parallel aliquots of bioassay samples immediately prior to analyses. Additionally, samples were screened for 1397 compounds with methods adapted from Bergmann *et al*. and Allan *et al*.^41,42^.

For the 62 PAH method, labelled internal surrogate standards of known concentration are used to quantify PAHs with similar physiochemical characteristics. Response ratios of surrogates to individual analytes were established using a nine-point calibration curve using standards ranging 10 to 1000 ng/mL. During analysis, continuing calibration verification (CV) samples of known concentration are typically run before and after every analytical batch and after every fifteen samples within large batches. Flanking C.V.s must meet data quality objectives (DQOs) for the analytical run to be considered valid. PAH method DQOs are defined as 80% of analytes being within 30% of the known value.

The Multi Analyte Screen Version 1530 (MASV15)^43^ analyte screening method uses deconvolution reporting software (DRS) coupled to a standard GC-MS method locked to the retention time of chlorpyrifos and an automated M.S. deconvolution and Identification system (AMDIS). As above, during analysis, DRS continuing calibration verification (CV) samples of known concentration are typically run before and after every analytical batch and after every fifteen samples within large sets. Flanking CVs must meet data quality objectives (DQOs) for the analytical run to be considered valid. DRS Method DQO’s are defined as retention time within 19.10 and 19.36 min, and positive detections between 80% and 120%. Each analyte detected by AMDIS is confirmed by the chemist using AMDIS peak selection and retention time match. At least one of the qualifier ion ratios is within the ratio established during analyte addition. Analytes measured are listed on the portal, but if there are no valid measurements they are not shown in the graph.

### Zebrafish measurements

Embryos are assessed at 24 and 120 hpf for mortality or malformations. Data is collected for each animal and a summary of affected and normal for each concentration per chemical is recorded. The experimental design includes a minimum of 24 animals being exposed per concentration. The average incidence rate per day (across all experimental plates) is ~12% (both morbidity and mortality). A minimum of 6 plates is conducted in a day to compute a daily global control incidence rate in addition to a chemical control incidence rate. In addition to the negative control, a daily positive control plate (ethyl parathion) is run per experimental day with an estimated effective concentration that induces 50% adverse effects (EC_50_) within 1 standard deviation. Therefore, to determine whether a chemical/sample causes developmental toxicity, both the global negative control and chemical negative control must have a lower than 20% morbidity rate. The positive control must induce the expected EC_50_ concentration. In the cases that the negative or positive quality control metrics are not met, the experiment is re-run.

### Zebrafish dose-response values

Determining the quality of dose-response measurements in high throughput quantitative data is still an open challenge. Toward this end, we provide objective and reproducible criteria as recommended by the United States Environmental Protection Agency^39^, described below.

Our workflow, available at http://github.com/PNNL-CompBio/srpAnalytics, evaluates quality control at various steps of the technical platform. Ultimately, data set quality control categorization consists of three distinct classes (Fig. 4).

Descriptions of each of the QC score classes are in Table 3. A QC score of’Poor’ is assigned when there are less than 3 dose groups, as there are not enough points to fit a curve, or when there are at least 3 doses, but no trend is detected in dose-response data, i.e. when experimental data shows a decreasing trend or flat response along with chemical concentration increase. Among the 6885 unique conditions (e.g. chemical ID-chemical concentration-endpoint combination), less than half (2781) had this designation.

**Table 3:** Description of concentration-response curve quality control scores for each benchmark dose (BMD) calculation made across endpoints, chemicals, and sample extracts.

Quality Control (QC) Score	Description	QC Badge on portal	Number of chemical BMDs	Number of extract BMDs
Poor	There are not enough concentrations for BMD derivation, or no trend was detected. Therefore, BMD calculation is not performed.		2781	338
Moderate	Data resolution can fit a curve, but BMD analysis is unreliable.		1952	534
Good	Good or very good concentration-response data (p-value for non-flat curve < 0.32)		2152	413

For those data for which we were able to calculate a concentration-response curve, we used a t-test to measure the significance of the mean first-order response differences. If the p-value equals or is less than 0.32 (a value determined via hundreds of trials), then we gave the fit a QC score of “Good”. If the p-value is greater than 0.32, but still BMD is calculable, then ‘Moderate’ QC score is given, as the fractional responses systematically cancel each other.

In summary, our program is reproducible with transparent criteria and a fully automated method. Specifically, it processes various ranges of numerical values of dose-response groups (Fig. 4) and determines the quality of the data, and benchmark dose statistics when the quality is sufficient.

## Usage Notes

To facilitate interaction with the data described in this resource, we built a public-facing website that summarized the data and allows scientists to query specific compounds or sites of interest at http://srp.pnnl.gov.

**Fig. 5:**
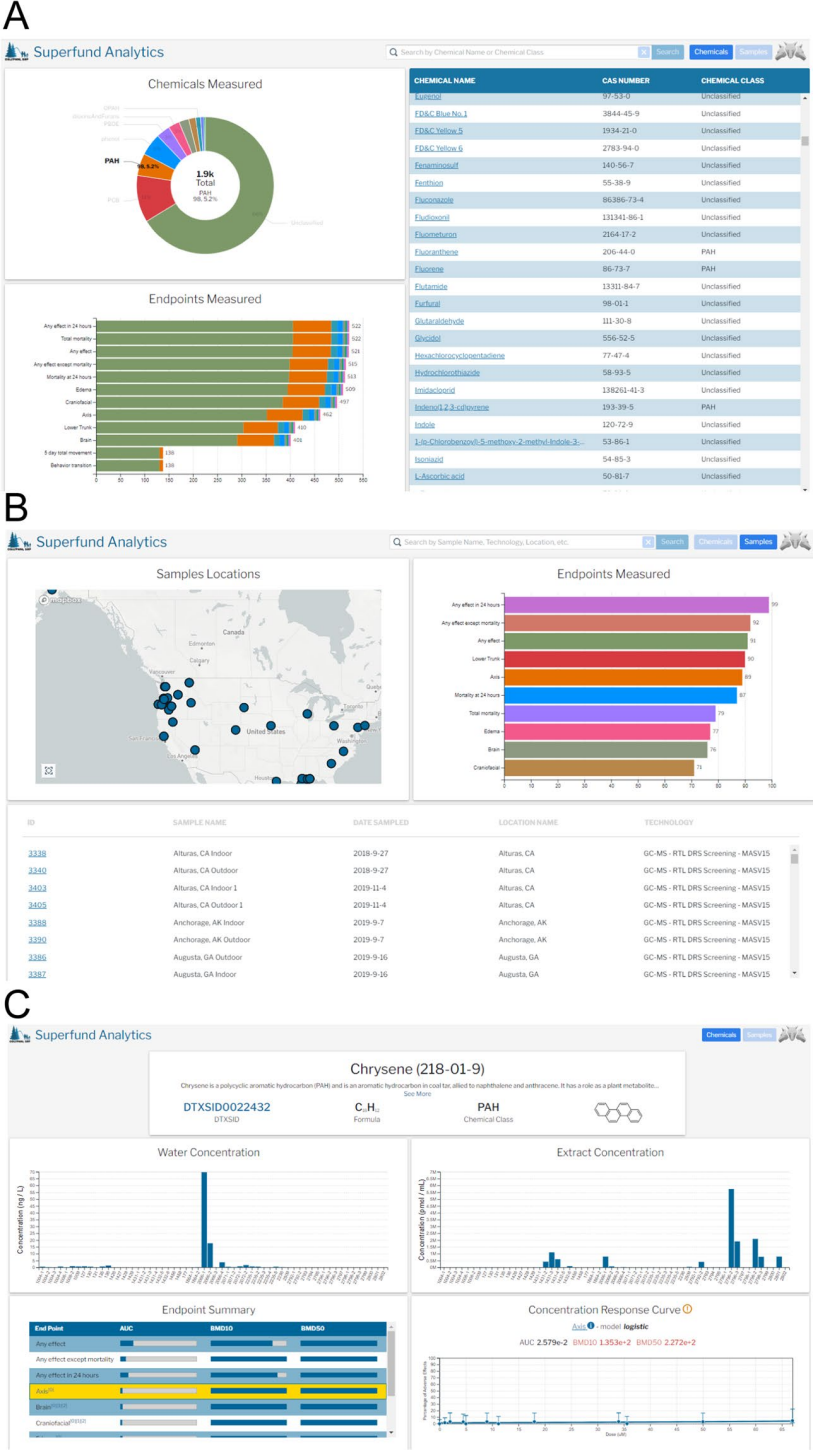
Images of SRP Analytics Portal public facing web site. The site can be entered by browsing chemicals or samples. (**A**) depicts the chemical summary page describing the total number of chemicals measured by class (upper left hand side), a table of all chemicals together with their CAS Id, and a summary of the end points. The table can be searched to filter by chemical class. (**B**) depicts the summary of environmental sample extracts, with a map of where they were collected, a list of their descriptions and the endpoints that were measured. (**C**) depicts the chemical page that describes a single chemical selected from panel (**A**). This includes its concentration across samples (top) and its effect in Zebrafish (bottom). A user can click on the sample name in the barplots to access sample information (or navigate from (**B**). Clicking on various endpoints changes the dose response curve values.

This website allows is comprised of three types of pages: a chemical summary page (Fig. 5A) that summarizes the chemicals samples and their representation in the data resource, a sample summary page (Fig. 5B) that summarizes the data for all environmental samples, and a details page that describes information about a specific chemical (Fig. 5C) or environmental sample.

The detail pages display the summary endpoints and concentration-response curves for each summary endpoint for which a curve could be calculated. The benchmark dose (BMD10, BMD50), and area under the curve (AUC) are depicted for each summary end point. They also show the chemical composition of samples when measured, and links out to Human gene expression data.

All data is freely available for download from PNNL’s DataHub resource^30^.

## Supplementary information

Supplementary Table 1

## Data Availability

All code required to analyse the data and format it for the package and web site are incorporated in a software container available at http://github.com/PNNL-CompBio/srpAnalytics or at Docker hub. To create a new data package with additional code, we can run the docker image with additional files in the prescribed data format. Command-line arguments to the docker will format the data to the schema on our github site so that it can be added to the repository.
