# Supplementary material for: The Superfund Research Program Analytics Portal: linking environmental chemical exposure to biological phenotypes
Source: Sci Data. 2023 Mar 21;10:151. doi: 10.1038/s41597-023-02021-5 (PMC10030892; doi:10.1038/s41597-023-02021-5)
Supplement: Supplementary file 1 — Supplementary Table 1 [file 41597_2023_2021_MOESM1_ESM.docx]

| **Endpoint labels** | **Short Name** | **Included in portal** | **Description** | **Ontology ID** |
| --- | --- | --- | --- | --- |
| MO24 | Mortality at 24 hours | Yes | Death occurred shortly after chemical dosing to 24 hpf. | ZP:0000306 |
| DP24 | Delayed Progress @ 24 hpf | No | Lag in development or otherwise morphologically different than control animals at 24 hpf. | ZP:0000305 |
| SM24 | Spon Movement @ 24 hpf | No | Frequency of spontaneous tail flexions at 28C (typically once every 15-20 seconds) is visibly faster or slower than control animals. | ZP:0009084 |
| MORT | Mortality at 5 days | No | Animals alive at the 24 hpf check found dead at the 120 hpf evaluation. | ZP:0000306 |
| CRAN | Craniofacial | Yes | Periocular edema, abnormally small or missing eye(s), misshapen snout and protruding jaw form a highly correlated suite of malformations simplified to ‘craniofacial defects.’ | ZP:0000943; ZP:0007203 |
| AXIS | Axis | Yes | The body axis is visibly curved, either concave or convex, where control animals are straight. | ZP:0005012 |
| EDEM | Edema | Yes | Distended clear region around the heart and/or yolk sac or top of the head with a lumpy appearance of swollen tissue. | ZP:0002060 |
| MUSC | Muscle | No | Swim bladder air bubble not present by 120 hpf, abnormal number of or disorganized chevron pattern of muscles, heart beat irregular or much different than 180 bpm, blood flow absent or slow in the common cardinal vein (duct of Cuvier) | ZP:0020238; ZP:000011 |
| LTRK | Lower Trunk | Yes | Shorter than normal body from the posterior of the yolk sac to the caudal fin, abnormal caudal fin | ZP:0003437; ZP:0010405 |
| BRN | Brain | Yes | Brain region abnormal in appearance based on color or size. | ZP:0001601; ZP:0000100; ZP:0008625 |
| SKIN | Skin | No | Hyper or hypo pigmented. | ZP:0100440 |
| NC | Notochord | No | Notochord sinuous instead of straight. | ZP:0000624 |
| TCHR | Touch Response | No | A gentle touch with a blunt tip probe produces no escape response. | ZP:0015467 |
| ANY_MORT | Any effect except mortality | Yes | Any morphological abnormality not including mortality |  |
| ANY120 | Any effect in 5 days | Yes | Any abnormality or death within 5 days (combination of all measurements at 24hr and 5 days) |  |
| ANY24 | Any effect in 24 hours | Yes | Any abnormality or death at 24hrs (aggregation of MO24, DP24, SM24 and NC24) |  |
| TOT_MORT | Total Mortality | Yes | Total mortality (aggregation of MO24 and MORT) |  |
| AUC1 | 5 day total movement | Yes | Behavior-total movement in dark minus total movement in light for the first light/dark cycle | ZP:0012599 |
| MOV1 | 5 day behavior transition | Yes | Behavior-transition from light to dark for the first light//dark cycle | ZP:0012599 |
